# Supplementary material for: The Relevance of Fetal Abdominal Subcutaneous Tissue Recording in Predicting Perinatal Outcome of GDM Pregnancies: A Retrospective Study
Source: J Clin Med. 2020 Oct 21;9(10):3375. doi: 10.3390/jcm9103375 (PMC7594040; doi:10.3390/jcm9103375)
Supplement: Supplementary file 1 [file jcm-09-03375-s001.pdf]

Journal of
Clinical Medicine

MDPI

Supplementary materials

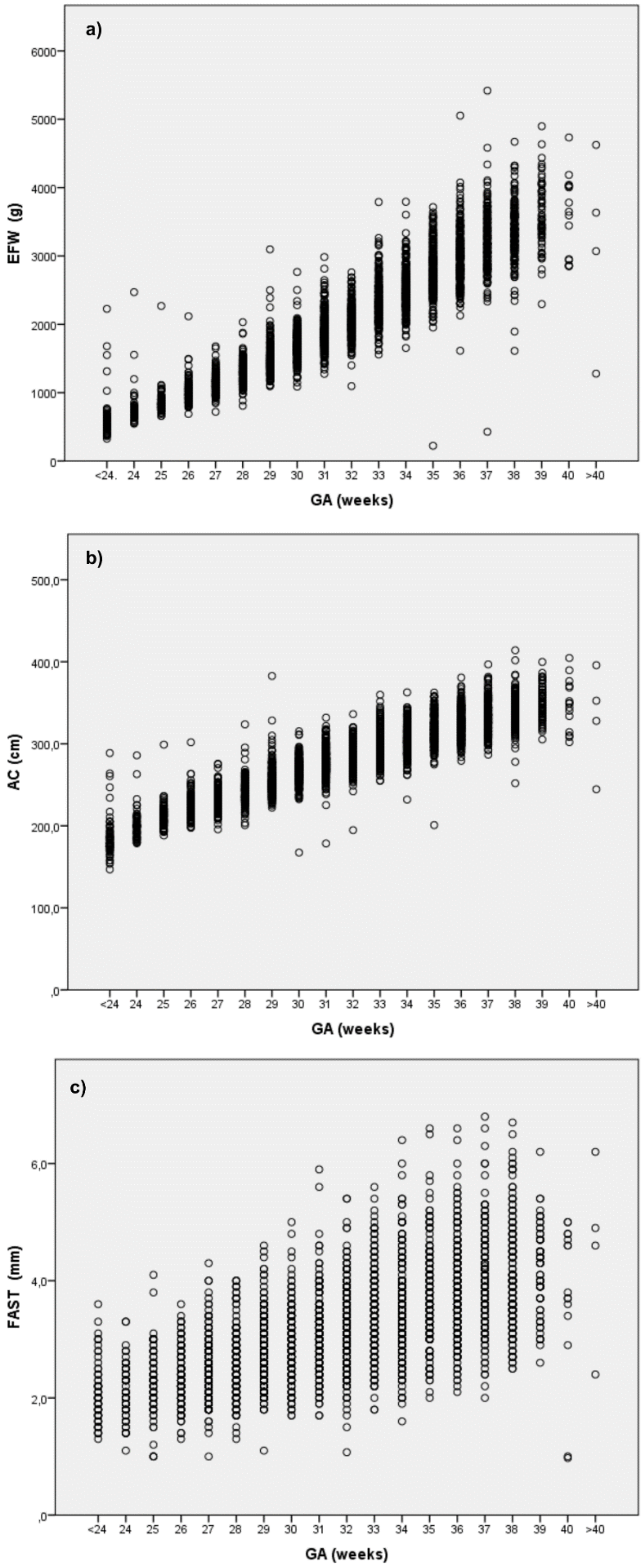

Figure 1. Scatter plots showing retrieved values of Estimated Fetal Weight (**a**) (EFW) Abdominal Circumference (AC) (**b**) and Fetal Abdominal Subcutaneous Tissue (FAST) (**c**) according to gestational age.
